# The Prevalence of Infertility and Loneliness among Women Aged 18-49 Years Who Are Living in Semi-Rural Areas in Western Turkey

**Published:** 2014-07-08

**Authors:** Mehmet Enes Gokler, Alaettin Unsal, Didem Arslantas

**Affiliations:** Department of Public Health, Faculty of Medicine, Eskisehir Osmangazi University, Eskisehir, Turkey

**Keywords:** Infertility, Loneliness, Prevalence, UCLA, Age

## Abstract

**Background:**

To determine the correlates and the prevalence of infertility in a group of women.

**Materials and Methods:**

This cross-sectional study was carried out on 570 subjects aged 18-49 years in a town of western Turkey between July and August 2012. Women who have inability to become pregnant despite regular sexual intercourse during the last year were considered to be infertile. UCLA Loneliness Scale was used to assess the severity of loneliness. The data were analyzed by Kruskal-Wallis, Mann Whitney U and Chi-square tests.

**Results:**

The mean age of the participants was 35.48 ± 8.39 years. The frequency of the infertility in our study was 12.8% (n=73). The prevalence of infertility was higher in those with a history of gynecological disease or gynecologic surgery and in those with menstrual irregularity (p<0.05; for each). The mean score on the UCLA Loneliness Scale was 32.16 ± 9.49 (from 20 to 70). In this study, no difference was found between the level of loneliness and who is responsible for infertility among infertile/fertile women (p≥0.05). Level of loneliness among the women with primary infertility was higher compared to the women with secondary infertility (p<0.05).

**Conclusion:**

The prevalence of infertility among the women was relatively high. It was concluded that prospective studies are needed in order to expose the relationship between the infertility and the level of loneliness in women.

## Introduction

Infertility is defined as the failure to achieve a pregnancy after at least 1 year of regular unprotected sexual intercourse (1).

The etiology of infertility is suggested to be related to a female factor in 25-40% of the cases and to a male factor in 40-55%. Unexplained infertility accounts up to 10% of the cases. The most common causes of infertility include the male factors such as sperm disturbance, female factors such as ovulation dysfunction and tubal disorders, both male and female factors, and unexplained infertility. Prevalence of infertility increases with the changes in living conditions over the years. Changing living conditions lead to increased tobacco use and alcohol consumption, aging of the population, stressful living conditions, and decreased physical activity-induced obesity, all of which are among the reasons that increase the prevalence of infertility (2).

Infertility affects 10-18% of married couples all over the world, and approximately, 72.4 million couples are estimated to be infertile (3). Previous studies from Turkey reported the prevalence of infertility as ranging from 3.2 to 20.0% (4-6).

Infertility is not only a gynecological disease but also an important health problem that has social, economic, cultural, and psychological effects. Infertility manifests itself as a sudden and unexpected life crisis, perhaps could not be explained, the diagnosis is delayed, and causes excessive stress and forcing adaptation that negatively affects the quality of life of couples (7). For that reason, infertility may have several emotional and psychological consequences on infertile couples.

It’s known that levels of anxiety and loneliness are higher among infertile women who are generally more negatively affected than their husbands (8, 9). As in many other populations, being a mother and raising children is very important for women in our population. Thus, women feel themselves empty, defective, inadequate and worthless when faced with the problem of infertility in later stages of their lives. This situation leads to loneliness by isolating themselves from their community.

This study aimed to evaluate the prevalence of infertility, to examine some possible factors associated with infertility and to assess the level of loneliness among married women aged 18-49 who were living in the district of Mahmudiye.

## Materials and Methods

This cross-sectional survey of women with infertility and of some characteristics particularly seen in patients with infertility was carried out in Mahmudiye, a rural district of a town in western Turkey. It was conducted on all the married women aged 18-49 years between July 5 and August 29, 2012. According to the Turkish Statistical Institute (TUİK), the total population of the semirural town, in which the study was carried out, was 4731. The total number of the women aged 18-49 years and living in the town was 1057. The number of those who are married was 824 (10). No sample selection was made in order to reach all of these women, and all women were visited at their home. Women who were at home and accepted to participate in the study after being informed about the study (n=570; 69.2%) comprised the study group.

The questionnaire consisted of two parts. The first part included the individuals’ socio-demographic characteristics (age, gender, education level, employment status, income level, family type, cigarette and alcohol habits, and obesity) and some of the factors thought to be associated with infertility (menstruation, dysmenorrhea, age at menarche, gynecological disease history, gynecologic surgery history, infertility type, duration of infertility, and individual who are responsible for the infertility) (11-15). The history of gynecological diseases or surgery was asked as "Do you have a history of a physician diagnosed disease related to women’s diseases?" or "Did you undergo any surgery related to women’s disease?". The second part of the questionnaire included the questions of UCLA Loneliness Scale for the assessment of the severity of loneliness.

Households in the town center were visited one by one during the study period. The study group consisted of a total of 570 women who had marriaged at least once, were at home and accepted to participate in the study.

Permission for the study was obtained prior to collection of data by contacting and receiving approval from the appropriate management authority, the health directorship of the city involved. Informed consent was obtained from the subjects participating in the study according to that established by the Ethical Principles for Medical Research Involving Human Subjects in the Helsinki Declaration (16). After informing the women about the purpose of the study, and where and how the data would be used, verbal consents were obtained. The pre-prepared questionnaire forms were filled by the researchers with the face-to-face interview method.

In our study, women who have inability to become pregnant despite regular sexual intercourse during the last year were considered to be infertile. Couples who have not ever become pregnant were evaluated as primary infertile and those who have been pregnant at least once, but never again were evaluated as secondary infertile.

In this study, UCLA Loneliness Scale was used to assess the level of loneliness. The scale has been developed in 1978 by Russell et al. (17) and reliability and validity studies for Turkish version of UCLA were performed by Demir in 1990 (18). Scale is composed of four-point likert-type of 20 items that contain 10 positive and 10 negative expressions. Total score that can be taken from the scale varies from 20 to 80. The level of loneliness increases with the increasing total score.

The data were analyzed using Statistical Package for the Social Sciences (SPSS; SPSS Inc., Chicago, USA) version 20. The statistical analysis was carried out using Kruskal-Wallis (KW), Mann Whitney U (U) and Chi-square tests (χ²). A value of p<0.05 was considered as statistically significant.

## Results

The mean age of the participants was 35.48 ± 8.39 years (18-49 age range). Of the women, 542 (95.1%) were married and 28 (4.9%) were widowed at that time. In our study, because the number of alcohol consumers was very low (n=2), analysis was not performed for this variable.

The frequency of infertility in our study was found to be 12.8% (n=73). The socio-demographic characteristics of the women with and without infertility are presented in table 1.

**Table 1:** The socio-demographic characteristics of the females with infertility and without infertility


Socio-demographics	Infertility			Statistical analyses
	No N (%)^a^	Yes N (%)^a^	Total N (%)^b^	χ²; p

**Age group**					
**18-24**	61 (91.0)	6 (9.0)	67 (11.8)	3.265; 0.659
**25-29**	72 (86.7)	11 (13.3)	83 (14.6)	
**30-34**	93 (86.9)	14 (13.1)	107 (18.8)	
**35-39**	102 (90.3)	11 (9.7)	113 (19.8)	
**40-44**	77 (83.7)	15 (16.3)	92 (16.1)	
**45-49**	92 (85.2)	16 (14.8)	108 (18.9)	
**Education level**					
**Illiterate**	84 (84.8)	15 (15.2)	99 (17.4)	0.590; 0.745
**Primary/secondary school**	292 (87.7)	41 (12.3)	333 (58.4)	
**High school and over**	121 (87.7)	17 (12.3)	138 (24.2)	
**Employment status**					
**Unemployment**	439 (86.6)	68 (13.4)	507 (88.9)	1.054; 0.305
**Employment**	58 (92.1)	5 (7.9)	63 (11.1)	
**Family income status**					
**Bad**	40 (87.0)	6 (13.0)	46 (8.1)	0.151; 0.927
**Fair**	366 (86.9)	55 (13.1)	421 (73.9)	
**Good**	91 (88.3)	12 (11.7)	103 (18.1)	
**Family type**					
**Nucleus**	393 (86.4)	62 (13.6)	455 (79.8)	1.017; 0.313	
**Large family**	104 (90.4)	11 (9.6)	115 (20.2)		
**Smoking**					
**No**	382 (87.8)	53 (12.2)	435 (76.3)	0.425; 0.515	
**Yes**	115 (85.2)	20 (14.8)	135 (23.7)		
**Obesity**					
**No**	392 (88.3)	52 (11.7)	444 (77.9)	1.737; 0.188
**Yes**	105 (83.3)	21 (16.7)	126 (22.1)	
**Total**	497 (87.2)	73 (12.8)	570 (100.0)	


a; Percent for the row and b; Percent for the column.

Of the women, 116 (20.4%) had a history of gynecological disease and 45 (7.9%) had a history of gynecologic surgery. Most women (80.9%) reported that they had regular menstruation. The frequency of dysmenorrhea was found to be 28.5% among the women menstruating. Some gynecological characteristics of women with/without infertility are given in table 2.

In this study, the numbers of women with primary and secondary infertility were 28 (38.4%) and 45 (61.6%), respectively. On the other hand, it was reported that 46.6% of infertile cases were female-related and 8.6% were male-related. Moreover, 45.2% of the infertile cases were unexplained infertility.

The mean score on UCLA Loneliness Scale was 32.16 ± 9.49 (from 20 to 70). The distribution of mean scores on UCLA Loneliness Scale according to some features of infertility is given in table 3.

**Fig 1:**
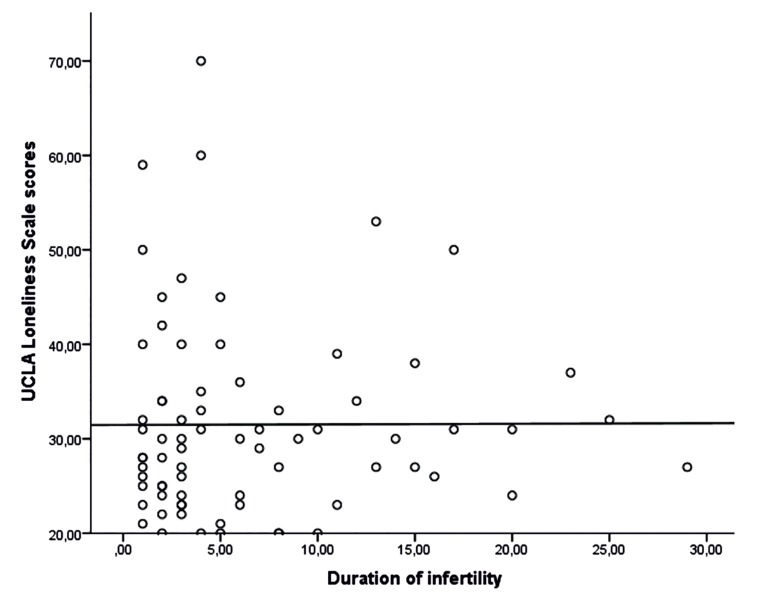
The distribution of UCLA Loneliness Scale scores obtained from the duration of infertility in women.

In our study, the mean duration of the infertility in women was 6.58 ± 6.41 (from 1-29) years. There was no relationship between the scores of infertile women on UCLA Loneliness Scale and the duration of infertility (rs=0,050; p=0,673). The distribution of the scores on UCLA Loneliness Scale according to the duration of infertility in women is given in figure 1.

**Table 2:** Some gynecological characteristics of women with/without infertility


Gynecological characters	Infertility			Statistical analyses
No N (%)^a^	Yes N (%)^a^	Total N (%)^b^	χ²; p

**Age at menarche (year)**				
**≤12**	132 (90.4)	14 (9.6)	146 (25.6)	6.217; 0.102
**13**	170 (86.3)	27 (13.7)	197 (3.6)	
**14**	103 (81.7)	23 (1.3)	126 (22.1)	
**≥15**	92 (91.1)	9 (8.9)	101 (1.7)	
**Menstrual regularity^¥^**				
**Regular**	392 (89.7)	45 (10.3)	437 (80.9)	5.715; 0.017
**Irregular**	83 (80.6)	20 (19.4)	103 (19.1)	
**Dysmenorrhea^¥^**				
**No**	340 (88.1)	46 (11.9)	386 (71.5)	0.000; 1.000
**Yes**	135 (87.7)	19 (12.3)	154 (28.5)	
**History of gynecological disease**				
**No**	411 (90.5)	43 (9.5)	454 (79.6)	20.785; 0.000
**Yes**	86 (74.1)	30 (25.9)	116 (20.4))	
**History of gynecologic surgery**				
**No**	468 (89.1)	57 (10.9)	525 (92.1)	20.484; 0.000
**Yes**	29 (64.4)	16 (35.6)	45 (7.9)	
**Total**	497 (87.2)	73 (12.8)	570 (100.0)	


a; Percent for the row, b; Percent for the column, ¥; The number of women who are menstruating

**Table 3:** The distribution of UCLA Loneliness Scale mean scores of the study group about some features of infertility


Some features of infertility	N	UCLA Loneliness Scale score Median (min-max)	Statistical analysesU/KW; P

**Infertility**			
**No**	497	30.0 (20.0-67.0)	1.074; 0.283
**Yes**	73	30.0 (20.0-70.0)	
**Total**	570	30.0 (20.0-70.0)	
**Infertility type**			
**Primary**	28	32.0 (20.0-70.0)	2.266; 0.023
**Secondary**	45	28.0 (20.0-50.0)	
**Total**	73	30.0 (20.0-70.0)	
**Wife responsible for infertility**			
**Female**	34	31.0 (20.0-60.0)	0.454; 0.797
**Male**	6	29.0 (20.0-59.0)	
**Unexplained**	33	28.0 (20.0-70.0)	
**Total**	73	30.0 (20.0-70.0)	


## Discussion

Infertility is a major health problem that is common among married couples and leads to medical, social, cultural, and psychological problems. In this study, the prevalence of infertility was found to be 12.8% (n=73). Studies from some countries have reported the prevalence of infertility to vary between 3.4 and 15.0% (2, 19-22). In Turkey, the prevalence of infertility varies between 3.2 and 20.0% (4, 6, 23). These differences in the prevalence of infertility could be attributed to the fact that studies have been made in different populations and different diagnostic criteria have been used.

Decreasing number and quality of oocytes in women with aging may reduce the possibility of fertilization, leading to increased frequency of infertility (24). The results of several previous studies also support these findings (12, 25). However, in our study, there was no difference between the age groups in terms of the frequency of infertility. There are also other previous studies reporting similar results (26).

The costs of the diagnosis and treatment of infertility is extremely high. It is easier to cover the expenses of the treatment of infertility for the couples with higher level of income. In the study group, there was no correlation between family income level and the frequency of infertility. Accordingly, Eren and Bayram have also reported no correlation between family income level and the frequency of infertility in their studies (12, 27).

In the extended family structure, strong family ties increase the responsibility towards the family. This represents an increased pressure on the individual and can lead to high stress which is a major risk factor for infertility. In addition, crowded family pattern may reduce the probability of fertilization by reducing the frequency of sexual intercourse. In this study, there was no difference between the women from a nuclear family structure and from a large family structure in terms of the frequency of infertility. There are also some other researchers finding no relationship between the family type and frequency of infertility (11, 27).

It has been reported that the frequency of infertility is high among smokers (15, 28). Some of the toxic substances contained in cigarettes, such as cotinine and cadmium, are considered to cause infertility by negative effects on the sperm production, movement, and morphology in men and by disrupting the follicular micro-environment and changing hormone levels during luteal phase in women (29). In our study, there was no difference between smokers and non-smokers in terms of the frequency of infertility. This result can be explained by that the infertile women in the study group might hide their smoking habit because of the feeling of guilt and the fear of getting a negative reaction from their environment and people providing medical assistance. Oğuz has also reported similar results (11).

One of the major effects of obesity in the body is the increased levels of insulin, leading to insulin resistance. Hyperinsulinemia increases androgen levels by reducing the levels of sex hormone-binding globulin, which can negatively affect the ovulation (30). For this reason, infertility is likely to be seen more frequently among obese women (26, 31). Some studies have also reported high frequency of infertility among obese women (32, 33). In this study, no differences were observed in terms of the prevalence of infertility between obese and non-obese women. This might be due to the similar dietary habits of women in the study group and to the efforts of infertile women to lose weight in order to be cured. Safarinejad has also found no relationship between obesity and the frequency of infertility in his study (28).

An early age at the first menstruation increases the incidence of diseases such as pelvic inflammatory disease that can cause infertility and spontaneous abortion at later ages (34). In our study, there was no relationship between the age of the first menstruation and the prevalence of infertility. Similar results have been reported in the study of Adamson et al. (15).

The major female-related causes of infertility are ovulatory disorders. If hypothalamus, pituitary and ovarian axis do not work appropriately, this can lead to ovulatory disorders such as anovulation, amenorrhea and menstrual disturbances. Many diseases such as polycystic ovary syndrome, hypothyroidism and hypothalamic-pituitary disorders that affect any stage of the axis are most likely to lead to infertility (35). In our study, the prevalence of infertility was significantly higher in women who had menstrual irregularity. Some studies have also reported similar results (11, 36). However, in a study by Helm et al. (32) it has been reported that there was no relationship between menstrual regularity/irregularity and infertility.

Dysmenorrhea is an important finding for many diseases such as polycystic ovary syndrome and endometriosis which are known to cause infertility. Therefore, the prevalence of infertility is likely to be high in women with a history of dysmenorrhea (37). However, in our study, no difference was found in the prevalence of infertility among women with and without a history of dysmenorrhea. This might be resulted from the small number of women in the study with a history of dysmenorrhea.

Gynecological diseases account for about 30-40% of all cases of female infertility (38). Both the direct effect of gynecological diseases and side effects of drugs used in the treatment can lead to infertility due to the disruption of reproductive function. In our study, the prevalence of infertility among the women with history of gynecological disease was found to be significantly higher than those without. Some previous studies have also reported that women with a history of gynecological disease have a higher prevalence of infertility (11, 28).

Structural changes in the genital organs of patients undergoing pelvic surgery can lead to infertility by preventing ovulation, fecundation, or implantation (39). In our study, prevalence of infertility was higher among women with a history of gynecological surgery. Similar results have also been reported in other studies (26, 28).

Gradual decrease in the number of oocytes from birth until menopause with no renewal and a reduction in the frequency of sexual intercourse with increasing age are known to decrease the fertility in women in older age (40). Infertility is one of the most serious problems that a person or couple can ever experience. The most common feelings of guilt, anger, frustration, and hopelessness often accompany the diagnosis of infertility (41). This may result in mental disorders such as anxiety, stress, depression, and loneliness in women, more frequently. In this study, no difference was found in terms of the level of loneliness among the infertile women with and without infertility. This might be resulted from the fact that these women may have more social support due to the presence of strong social relationships between people in the study region.

Primary infertile women feel more defective and incomplete because of having no births. Therefore, women with primary infertility are likely to feel more alone than those with secondary infertility. In our study, the level of loneliness was significantly higher among women with primary infertility than those with secondary infertility. It is well-known that mental disorders such as depression and loneliness are more common in infertile women than their husbands. In the study group, no difference was found between the level of loneliness and the individual who was responsible for infertility.

In the process of infertility, couples feel hopeless, failed and disappointed about having children every month. The repetition of this cycle may lead to feel more lonely and desperate (8, 9). Similar results have been reported in the studies of Kavlak and Saruhan (41). In the study group, there was no correlation between the duration of infertility and the level of loneliness. This result may be due to the acceptance of the infertility by women and their husbands over time.

The major limitations of this study are that it was a cross-sectional study, that it included only a single town, and that loneliness was not evaluated by precise diagnostic methods.

## Conclusion

In this study, infertility was found to be a common health problem among the married women. The prevalence of infertility was higher among women with menstrual disorders and in those with a history of gynecological disease or gynecological surgery. There was no difference between infertile and fertile women in terms of the level of loneliness. Whereas, the higher levels of the loneliness has been found among the women who have a primary infertility problem. It would be useful for the women to be informed about the causes and solutions of infertility, and those with infertility should be referred to a tertiary center. It was concluded that prospective studies are needed in order to expose the relationship between the infertility and the level of loneliness in women.
